# Antimicrobial and anti-aflatoxigenic activities of nanoemulsions based on *Achillea millefolium* and *Crocus sativus* flower extracts as green promising agents for food preservatives

**DOI:** 10.1186/s12866-023-03033-2

**Published:** 2023-10-07

**Authors:** Feriala A. Abu Safe, Ahmed N. Badr, Mohamed G. Shehata, Gharieb S. El-Sayyad

**Affiliations:** 1 Botany Department, Faculty of Women for Art, Science, and Education, Ain Shams University, Cairo, Egypt; 2 Food Toxicology and Contaminants Department, National Research Centre, Cairo, 12622 Egypt; 3 Department of Food Technology, Arid Lands Cultivation Research Institute, City of Scientific Research and Technological Applications (SRTA-City), New Borg El-Arab, 21934 Egypt; 4 Microbiology and Immunology Department, Faculty of Pharmacy, Ahram Canadian University (ACU), 6th October city, Giza, Egypt; 5 Department of Microbiology and Immunology, Faculty of Pharmacy, Galala University, New Galala City, Suez Egypt; 6 Drug Microbiology Lab, Drug Radiation Research Department, National Center for Radiation Research and Technology (NCRRT), Egyptian Atomic Energy Authority (EAEA), Cairo, Egypt

**Keywords:** Antibacterial, Anti-aflatoxigenic, Nanoemulsions, *Achillea millefolium*, *Crocus sativus*, Food preservation

## Abstract

**Background:**

Although the mechanism of action of nanoemulsion is still unclear, the modern use of nanoemulsions made from natural extracts as antimicrobial and anti-aflatoxigenic agents represents a potential food preservation and a safety target.

**Methods:**

Two natural nanoemulsion extracts of *Crocus sativus* (the saffron flower) and *Achillea millefolium* (the yarrow flower) were produced in the current study using a low-energy method that included carboxymethylcellulose and Arabic gum. The synthesized nanoemulsion was fully identified by different analytical methods. Detection of the volatile content was completed using GC-MS analysis. The antioxidant potential, and phenolic compounds content were analyzed in the extractions. The synthesized nanoemulsions were screened for their antimicrobial potential in addition to their anti-aflatoxigenic activity.

**Results:**

The droplet size of Saffron flowers was finer (121.64 ± 2.18 nm) than yarrow flowers (151.21 ± 1.12 nm). The Zeta potential measurements of the yarrow flower (-16.31 ± 2.54 mV) and the saffron flower (-18.55 ± 2.31 mV) both showed high stability, along with low PDI values (0.34–0.41). The nanoemulsion of yarrow flower revealed 51 compounds using gas chromatography-mass spectrometry (GCMS), with hexanal (16.25%), *β*-Pinene (7.41%), *β*-Myrcene (5.24%), D-Limonene (5.58%) and Caryophyllene (4.38%) being the most prevalent. Additionally, 31 compounds were detected in the saffron nanoemulsion, with D-limonene (4.89%), isophorone (12.29%), 4-oxy isophorone (8.19%), and safranal (44.84%) being the most abundant. Compared to the nanoemulsion of the yarrow flower, the saffron nanoemulsion had good antibacterial and antifungal activity. Saffron nanoemulsion inhibited total fungal growth by 69.64–71.90% in a simulated liquid medium and demonstrated the most significant decrease in aflatoxin production. Infected strawberry fruits coated with nanoemulsion extracts exhibited high antimicrobial activity in the form of saffron flower and yarrow flower extract nanoemulsions, which inhibited and/or controlled the growth *of Aspergillus* fungi. Due to this inhibition, the lag phase was noticeably prolonged, the cell load decreased, and the stability time increased.

**Conclusion:**

This study will contribute to expanding the theoretical research and utilization of nanoemulsions as green protective agents in agricultural and food industries for a promising protection from the invasion of some pathogenic bacteria and fungi.

## Background

Since ancient times, people have successfully treated illnesses using medicinal herbs. Because of their short half-lives and poor bioavailability profiles, many bioactive components found in herbs provide some health advantages but have limited therapeutic potential. The molecules from plants may have either a hydrophilic or a lipophilic character [1]. The poor absorption of highly hydrophilic bioactive via lipid membranes caused a decrease in biological effectiveness and pharmacokinetics [2]. The poor membrane permeability of the plant bioactive chemicals was another factor that restricted their potential therapeutic usage, as these compounds may have enormous molecular sizes. Of these herbals, *Achillea millefolium* and *Crocus sativus* are distinguished for their minor component content and bioactivity [3].

*Achillea millefolium* has a long history of utilization for treating inflammation, illness remedies, wound curing, and respiratory diseases. *Achillea millefolium* was also used in herbal tea mixes and phytopharmaceuticals. Previous studies revealed that *Achillea millefolium* extract has anticancer, antibacterial, anti-inflammatory, and antioxidant activities [4]. Saffron is one of the most valuable flowers produced in the Middle East. The components of Saffron are frequently utilized in processing food and nutraceutical applications for their bioactive compounds. Saffron flowers are recognized for flavoring, coloring, and taste-introducing natural additives [5]. Crocins, crocetin, safranal, picrocrocin, essential oils, minerals, and even tiny levels of vitamins B1 and B2 are the bioactive present in Saffron [6].

The bioactive components in Saffron are primarily responsible for antioxidant, anti-carcinogenic, anti-inflammatory, anti-tumor, and anti-depressant activities. As mentioned earlier, the body must be tactfully introduced to all of the bioactive to get optimized. A combination of internal and environmental factors determines whether or not bioactive components reach their intended target tissues in the human body [7].

Otherwise, food production’s harmful conditions include microbiological and fungal infections, particularly toxigenic fungi. These fungi and their related toxins can destroy food safety and food security. Fungal growth on food materials leads to tissue decay and food bulk loss [8].

Mycotoxins also turn food materials into culling food, which can not be exploited in future utilization. Based on their fluorescence under UV light, the four primary aflatoxins are designated AFB_1_, AFB_2_, AFG_1_, and AFG_2_. Aflatoxin B_1_ (AFB_1_), is the natural carcinogen with the greatest potency and is frequently the predominant aflatoxin formed by toxigenic fungal strains. However, different aflatoxins have been described, most prominently as the outcomes of mammalian biotransformation of the primary metabolites [9].

Plant bioactive components are restricted in benefits by their nutraceutical applications due to their insufficient permeability, absorption, and solubility. This factor has become the most significant barrier to efficient herbal bioactive utilization [10]. The transformation of herbal extracts into the nanoemulsion form may provide a stable solution of nanocarriers, which may entrap active moieties inside the core of excipients, which has the potential to mitigate these shortcomings and provide a solution [11].

This study aimed to elucidate the antimicrobial and anti-aflatoxigenic effects of nanoemulsions based on natural extracts from *Achillea millefolium* and *Crocus sativus* flowers, which will contribute to expanding the theoretical research and utilization of nanoemulsions as green protective agents in agricultural and food industries.

## Materials and methods

### Chemicals

Sigma-Aldrich Chemical Co. (St. Louis, MO, US) provided all of the chemicals, microbiological requirements, and media. All of the solvents and compounds were of investigative chromatographic quality.

### Microorganisms

*Bacillus cereus* EMCC 1080, *Staphylococcus aureus* ATCC 13565, *Listeria monocytogenes* ATCC 19111, and *Enterococcus faecalis* ATCC 33186 as four Gram-positive bacterial strains, *E. coli* ATCC 51659, *Salmonella enterica* ATCC 10708, *Pseudomonas aeruginosa* NRRL B-272, and *Klebsiella pneumoniae* LMD 7726, as four Gram-negative bacterial strains were applied for the antibacterial investigation. The DSMZ collection of microorganisms (Leibniz Institute DSMZ-German Collection of Microorganisms and Cell Cultures, Braunschweig, Germany) provided these isolates. They were subsequently preserved for 24 h at 37 °C on nutrient agar slants, and then stored at 4 °C until application.

Five species of toxic fungus obtained from the agro-food microbial culture collection (ITEM), ISPA, CNR, Italy, were subjected to an antifungal test. *Candida albicans* ATCC 18804, *Aspergillus flavus* ITEM 698, *Aspergillus parasiticus* ITEM 11, *Penicillium verrucosum* NRRL 695, and *Fusarium graminearum* ATCC 56091 were the different fungi that were present. Fungal organisms were preserved on a Czapek-dox medium before the evaluation test.

### Statement

The Institutional Review Board of the National Research Centre (NRC) approved the use of HeLa cells in this study. All experiments followed the guidelines set forth by the National Institutes of Health and the Declaration of Helsinki for biomedical investigation including human subjects.

### Plant material

The plants of *Crocus sativus* (the saffron flower) and *Achillea millefolium* (the yarrow flower) were purchased and identified by the Herbarium of the National Research Centre, Cairo, Egypt. The plant was collected from the Institution farm at a longitude of 30° 4’ 36”, and a latitude of 30° 39’ 59”. *Crocus sativus* (the saffron flower) and *Achillea millefolium* (the yarrow flower) have been used as plant materials. The dried flowers were ground into a fine powder before extraction.

### Raw materials extraction

*Crocus sativus* (the saffron flower) and *Achillea millefolium* (the yarrow flower) raw materials were extracted using an environmentally safe solvent solution consisting of aqueous isopropyl (80%). Using an ultrasonic probe with the following settings: amplitude 45%, frequency 80 kHz, duty 60%, duration 40 min, and temperature 20 °C, milled powder was sonicated in a 1: 4 (v/v) isopropyl solution. Using a lab lyophilizer (FD-10-MR Malti-manifold, Esquire Biotech, India), the extracts were concentrated and lyophilized. This procedure was used to get the extracted solutions ready for the tests that followed.

### Analysis of the total phenolic and total flavonoid comfortable

The total phenolic content of the samples was determined using the Folin-Ciocalteu reagent. By comparing them to a control sample, the approach used by Badr et al. [12] quantified them in milligrams of Trolox equivalents per gram of dehydrated weight (mg GAE/g DW). With the use of a UV spectrophotometer (Shimadzu, Kyoto, Japan), absorbance at 760 nm was recorded. The total flavonoid content of the extracts was determined using the procedures described by Shehata et al. [13]. The results were expressed as milligrams of quercetin equivalent per gram of dehydrated weight (mg QE/g DW).

### Antioxidant evaluation

Three separate assays (DPPH, ABTS, and FRAP) were used to measure the antioxidant activities of the two types of extracts, following the methodology described by Abdel-Razek et al. [14]. The biological activity of the extract, primarily as an antibacterial and shelf-life extension agent, was evaluated using the antioxidant activity as a guide. Micrograms of Trolox equivalents per gram of dry weight extract (µM TE/g DW) were used to assess the antioxidant capacity.

### Detection of the volatile content using GC-MS analysis

The gas chromatography (Agilent 8890 GC System), mass spectrometer (Agilent 5977B GC/MSD), and fused silica capillary column (30 m, 0.25 mm i.d., 0.25 mm film thickness) were utilized to analyze the hydrodistilled EOs. Initially set at 50 °C, the oven’s temperature was then programmed to increase by 5 °C per minute to 220 °C then by 10 °C per minute to 280 °C. The injection temperature was 250 °C and the carrier gas was helium, flowing at a rate of 1 mL/min. The mass spectra in the electron impact mode (EI) had scan m/z values that varied from 39 to 500 amu at 70 eV. By comparing them to values from the mass spectra library, the separated peaks were identified.

For HS-SPME extraction, 1 g of the floral extract powders was put into a 20-mL headspace vial produced by Gerstel, Mülheim a/d Ruhr, Germany, with a magnetic crimp lid and septum. The 50/30 m DVB/CAR/PDMS divinylbenzene/carboxy/polydimethylsiloxane fiber from Supelco, Bornem, Belgium, was used for a direct sample of volatiles for 60 min at 30 °C, and the fiber was desorbed for 2 min at 250 °C in the GC-MS inlet. SPME extraction and desorption were carried out by an automated multipurpose sampler (MPS-2, Gerstel).

### Determination of the fractions of phenolic compounds

The phenolic fractions of the extracted materials were calculated using the technique developed by Stuper-Szablewska et al. [15]. The phenolic content at 280 and 320 nm was determined by comparing the retention periods of the analyte peaks with the added standards. The results had a quantification limit of 10 ng/g of material, and they were computed in triplicate and reported as means ± SEM.

### Preparation of nanoemulsion form applied extracts

The extracts were concentrated in powder form and were used to create the nanoemulsion. The methodology of Sundararajan et al. [16] was modified to apply the concentrated extract to create the nanoemulsion. Briefly, nanoemulsion quantification was developed using a low-energy method consisting of 35% carboxymethyl-cellulose (CMC) solution (2%, w/v), 15% Arabic gum (AG) solution (3%, w/v), 25% extract solution (10 mg in aqueous ethanol), 10% polysorbate 80, and 15% glycerol at a total volume of 200 mL [17].

The extract (10 mg) was first dissolved in 50 mL ethanol (50%, v/v), then the polysorbate 80 was added before the mixture was stirred (800 rpm/2 h) using magnetic stirring. The aqueous solution of the CMC was prepared by dissolving 2 g in 100 mL distilled-warm water and stirred using a magnetic stirrer for 1 h till completely dissolving. The solution of the AG was ready by dissolving 3 g of powder into 100 mL of distilled water and then stirring for 1 h up to the complete dissolving. The targeted quantities of CMC and AG solutions were then mixed by stirring for more than 1 h, and then the resulting solution was added dropwise at a flow rate of 1 mL/min to the coarse extract-polysorbate prepared before. The mixture was stirred (1200 rpm /10 h) til the staple coarse was formed. The nanoemulsion was then kept at room temperature (20 °C) and measured for stability.

### Particle size and Zeta potential

The Zeta sizer Nano-ZS measures the zeta potential of a sample of particles using dynamic light scattering (DLS). To measure the particle velocity in an applied electric field of a specified value known as electrophoretic mobility, a laser is sent through the sample in this procedure. Additionally developed was the polydispersity index (PDI), which depicts the dispersion of nanoparticle particle sizes. Using a dynamic light scattering device (Nano ZS, Malvern Instruments, Worcestershire, UK), the particle size and zeta potential were assessed. To determine the particle size, the emulsion sample (2 drops) was diluted in water (2 mL) and transferred to a cuvette. The material for the zeta potential measurement was contained in a capillary cell (25 µL), and it was then diluted in water (2 mL). To determine the particle size as a Z-average, the Stokes-Einstein relation and its accompanying polydispersity index (PDI) were utilized. For each sample, zeta potential and particle size were evaluated three times.

### Viscosity measurement of nanoemulsion

The viscosity of the nanoemulsions was measured throughout storage. The viscosity values were calculated as the average of 5 days of storage time measurements. A viscometer from Brookfield Engineering Laboratories (DV-E Model, Middleboro, MA, USA) outfitted with a spindle-type measuring system (CPA-40Z Viscometer) was used to assess the viscosity. The samples were moved to the apparatus and acclimated for 5 min at 25 °C before measurement. The daily measurement was done three times, and the mean value over five days was determined by adding all the recorded values.

### Titrable acidity

Titratable acidity was determined as a percentage of citric acid by titrating 10 mL of the extract with NaOH (0.1 N) solution to pH 8.1. The pH was measured using a pH meter (GenWay, pH 2001; Genway Instruments, UK).

### Antibacterial activity

The minimum inhibitory concentration (MIC) and diffusion assays of resistance were used to analyze the antibacterial action. Gram-negative and Gram-positive bacteria strains were utilized to examine the extracted materials. The lowest dilution that prevented bacteria from growing matched to controls was the minimal inhibitory concentration. The previously described method [18] was employed to calculate the MIC results for the strains. The tests were conducted using the CLSI M7-A6 reference procedures for bacteria developed by the Clinical and Laboratory Standards Institute. The extracts were dissolved in DMSO at a wide range of concentrations, ranging from 0.05 to 1000 µg/mL. Using a microplate reader (ASYS UVM 340, Cambridge, UK), the MIC was calculated by measuring each well and using the results. The tested strains mentioned in the technique given were used in the well-diffusion test [12]. Chloramphenicol (at a concentration of 50 µg/g) was utilized as a standard antibiotic.

### Antifungal activity

The minimum fungicidal concentration (MFC) and the simulated growth medium were employed as two metrics to evaluate the antifungal action. The two experiments against different fungus assessed the extracts. Itraconazole, at a dosage of 25 g mL^− 1^, was utilized as a control antifungal for the applicable fungal strains in accordance with the Clinical and Laboratory Standards Institute (CLSI) fungus reference methods CLSI M27-A3 [18]. However, Shehata et al.‘s earlier research [19] using the Czapek Dox broth medium allowed for the examination of the extract’s impact on the pace of mycelia formation.

### Cytotoxicity and anti-cytotoxic effect

The normal hepatic HL-7702 cell line strains were cultured in DMEM growth medium that was enhanced with antibiotic treatment (0.9% saline with 20 mg amoxicillin and 25 mg chloramphenicol), 10% phosphate saline (PBS), at a density of 1 × 10^4^ cells/well (100 uL). They were then held at 37 °C and 5% CO_2_ for the remainder of the day [20]. Immediately after the attachment, HL-7702 cells were subjected to extract dilutions ranging from 1.000 to 6.25 g/mL. Following that, 10 uL of a 12-mM MTT stock solution containing 5 mg/mL MTT in sterile PBS was individually applied to each well. The MTT solution was destroyed after 4 h of storage (at 37 °C). In comparison to the extracts that were given, the cells’ vitality was evaluated. The experiment was designed in 5 groups consisting of the positive control (5-fluorouracil), treatment 1 (yarrow nanoemulsion), treatment 2 (yarrow extract), treatment 3 (Saffron nanoemulsion), and treatment 4 (Saffron extract).

### Anti-aflatoxigenic impact of the extract

We investigated the effects of the extract on the poisonous fungus strains of *A. flavus* ITEM 698 and *A. parasiticus* ITEM 11. At the concentration of 1 mg extract/100 mL medium, the antifungal activity was evaluated to suppress fungal growth and aflatoxin production. The 10^5^ spores/mL dilution was suspended in 250 mL of yeast extract sucrose (YES) in a 1 L conical flask. The control flasks (extract-free) and treatments were held for 5 days at 22 °C and 12 days at 28 °C, respectively, to measure growth change. The experiment was performed three times, and statistics were run.

### Mycotoxin extraction and determination

The AFB_1_, AFB_2_, AFG_1_, and AFG_2_ aflatoxin concentrations were tested in the medium growth-collected solutions. The AFs were removed from the samples using the same method as Ertekin et al. (2016). A blender was used to combine sodium chloride (5 g), methanol-water (100 mL; 80%), and exactly 50 mL of the growth medium for one minute at high speed. Before the collected solution was filtered once again, the combination was filtered using a Whatman No. 1 filter using an Aflatest® immunoaffinity column (VICAM, Watertown, MA, USA). The peristaltic pump (Golander BT100S pump, Dechant-Heimbach-Str., Bonn 53,177, Germany) and chromatographic manifold unit (SPE Vacuum Manifold Chromabond®, Roth Carl, Karlsruhe, Germany) were connected, and the rate was set to collect the filtrate at a rate of 1 drop per second.

Then, as part of the cleaning procedure, the column was cleaned twice with distilled water. Following cleaning, 1 mL of the aflatest® developer was added to the methanol- and methanol-based (1 drop per second) aflatoxin extraction from the column. Higher amounts of contamination in diluted samples were quantified. The eluted fraction’s fluorescence was measured using VICAM equipment after being diluted twice with HPLC water (VICAM Series). Every procedure step was carried out following the manufacturer’s guidelines. [21].

### Application of nanoemulsion on strawberry fruits

Nanoemulsion was applied to save the packed fruit and shelf life extension. Strawberry fruits (*Fragaria ananassa* cultivar Red Merlin) were purchased from local markets near Cairo governorate; the Longitude was 31° 24’ 22” and the latitude of 30° 12’ 86”.

According to the methodology of Filho et al. [22], the emulsion was applied to coat the strawberry fruits with modifications. Five groups were designed for each extract: the control, yarrow extract, Saffron extract, yarrow nanoemulsion, and Saffron nanoemulsion. The extract was applied for coating at 10 mg /mL of autoclaved water. Nanoemulsion and extract solution were utilized as 5 mL to treat 50 g fruits, then packed in sealed plastic bags. The bags were stored in the refrigerator after it was inoculated by 1.3 × 10^2^ CFU/mL of *Aspergillus flavus* spores. The total count was recorded for each group during 21 days of storage and expressed as log CFU/g. The less group in the CFU/g of strawberries reflects the enhancement of shelf life.

### Statistical evaluation

Each test’s outcomes were duplicated three times, and the data were shown as means ± standard deviation. SPSS V.16, statistical software for the social sciences, was used to analyze the data. Duncan’s multiple range test (p = 0.05) and ANOVA were performed to evaluate whether there was a significant difference between the mean readings.

## Results and discussion

### Determination of bioactive compounds

Numerous studies have shown that phenolic compounds and antioxidant activity have close ties to one another. It is believed that polyphenols’ lowering abilities as hydrogen- or electron-donating agents are connected to their propensity to serve as chemical free radical scavengers [23]. Secondary metabolites, also known as phenolic chemicals, are generated in plants and offer a variety of biological benefits for human health [24, 25]. Compounds found in medicinal plants such as phenolic acids, carotenoids, flavonoids, vitamins, and many more influence how organisms operate and interact with reactive oxygen species.

**Fig. 1:**
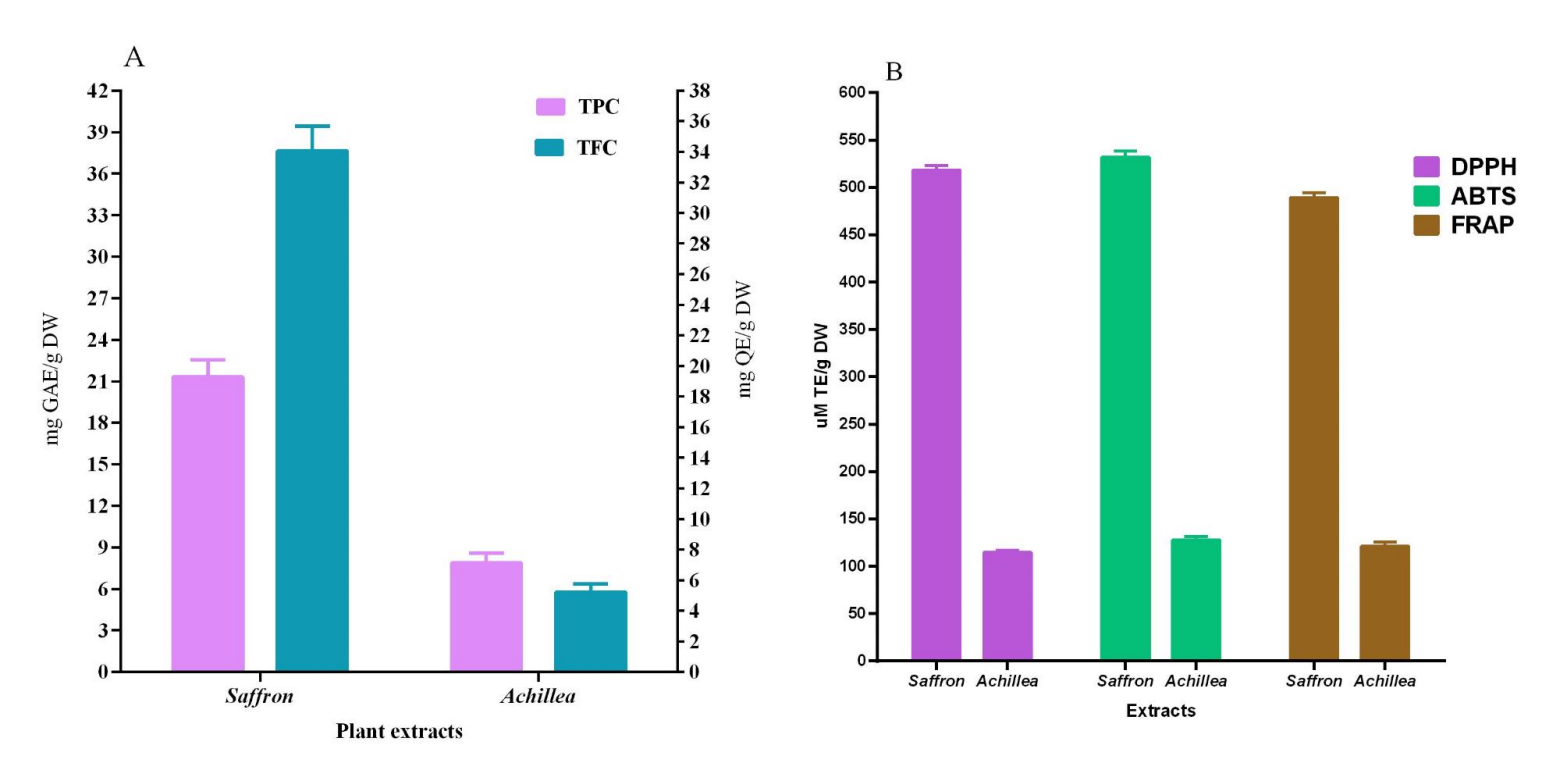
Total phenolic, total flavonoid contents and antioxidant activity of Achillea and Saffron extracts **A:** Total phenolic and flavonoid contents of the extracts gained from saffron and Achillea flowers; **B:** Antioxidant activity of the extracts from saffron and Achillea flowers determined by three assays (DPPH, ABTS, and FRAP)

The two plant extracts had significantly different total phenolic and flavonoid content (Fig. 1A). The analysis showed that the saffron flower extract had a higher total flavonoid content than the yarrow flower extract, which was (34.05 ± 1.64 mg QE/g DW) and(5.21 ± 0.56 mg QE/g DW) respectively. This result was confirmed when looking at the extracts’ total phenolic content (TPC). In the saffron extract, the TPC value was 21.31 ± 1.22 mg GAE/g DW, compared to 7.88 ± 0.69 mg GAE/g DW in the yarrow flower extract. These findings demonstrate the distinctive phenolic compound content of saffron flowers. The amount of phenolic and flavonoid compounds in the extracts of yarrow and saffron flowers was higher than that of natural herbs and spices like caraway, oregano, and rosemary. The spices’ total flavonoid concentration ranged from 3.24 mg of quercetin equivalent (QE) per gram of thyme to 0.38 mg of QE per gram of coriander [26]. As a result, plant phenolic and flavonoid compounds are crucial phytochemicals for human nutrition.

The antioxidant activity was assessed using three separate assays (Fig. 1B). The extract from saffron flowers appears more active based on the recorded results. Among the applied assays, the ABTS radical scavenging was recorded by a value equal to 531.66 ± 7.05 µM TE/g DW of Saffron flower extract. This value was 518.28 ± 5.17 and 489.36 ± 5.02 µM TE/g DW by DPPH scavenging and FRAP assays, respectively. The extract obtained from yarrows flowers showed lower antioxidant activity; the value was recorded at 127.66 ± 4.29, 121.47 ± 3.86, and 114.56 ± 2.25 µM TE/g DW for ABTS, FRAP, and DPPH assays of the scavenging activity, respectively. Inhibiting the etiology of dementia in mammalian cells, polyphenols work as exogenous chain-breaking antioxidants to reduce free-radical-mediated damage brought on by hazardous chain reactions in neuronal cells [27, 28]. These results suggest that Saffron and yarrow flowers can be used as natural antioxidants.

### Phenolic compound profile (RP‑HPLC)

Phenolic compounds, represented by phenolic and flavonoid fractions, were determined in the extracts obtained from the yarrows and saffron plants’ flowers (Table 1). The majority of phenolic acids recorded were chlorogenic (394.33 ± 2.47 µg/g DW), followed by p-coumaric (211.54 ± 2.74 µg/g DW) and sinapic (202.41 ± 1.28 µg/g DW). Rosmarinic and resveratrol phenolic acids were not detected in the yarrow flower extract. On the other hand, for the extract obtained from Saffron, ferulic acid (529.05 ± 2.34 µg/g DW), salicylic acid (328.22 ± 1.05 µg/g DW), rosmarinic acid (309.27 ± 2.51 µg/g DW), and caffiec acid (267.23 ± 1.37 µg/g DW) was recorded as the dominant phenolic acid. In contrast, 2-hydroxy benzoic acid was not detected in the Saffron extract.

**Table 1:** Phenolic fraction content determined in yarrows and saffron extracts obtained from the flower part of the plant

Concentrations (µg/g DW)					
Phenolic acids	Yarrows	Saffron	Flavonoid compounds	Yarrows	Saffron
Gallic	31.28 ± 1.16	87.16 ± 1.05	naringenin	212.36 ± 1.1	ND
4-hydroxy benzoic	1.37 ± 0.88	91.7 ± 1.81	naringin	ND	117.16 ± 1.22
2-hydroxy benzoic	147.21 ± 2.08	ND	catechin	ND	67.16 ± 1.12
chlorogenic	394.33 ± 2.47	95.21 ± 0.45	Epicatechin	ND	258.11 ± 2.88
vanillic	51.28 ± 1.17	121.37 ± 1.02	Myricetin	52.17 ± 1.37	59.22 ± 1.07
caffiec	159.34 ± 1.67	267.23 ± 1.37	Hesperidin	7.81 ± 1.44	8.23 ± 1.18
syringic	182.16 ± 1.81	137.11 ± 2.08	Quercetin	11.94 ± 1.56	421.51 ± 1.05
*p-*coumaric	211.54 ± 2.74	71.28 ± 0.81	Luteolin	131.16 ± 1.91	1.74 ± 0.22
sinapic	202.41 ± 1.28	55.08 ± 1.16	Kaempferol	15.86 ± 2.66	64.17 ± 0.55
Ferulic	137.51 ± 2.08	529.05 ± 2.34	Apigenin	14.87 ± 1.05	5.66 ± 0.37
cinnamic	8.41 ± 1.41	115.22 ± 1.54	Rutin	94.81 ± 1.27	7.05 ± 0.67
salicylic	3.27 ± 0.32	328.22 ± 1.05	Pyrogallol	ND	88.71 ± 1.94
Rosmarinic acid	ND	309.27 ± 2.51	Luteolin-7-Oglucoside	ND	14.37 ± 1.08
Resveratrol	ND	81.6 ± 1.85	Apigenin-7O-glucoside	ND	11.27 ± 0.97

The data were expressed as means ± SD (where n = 3); ND: represents the compounds that were not detected at the detection limit. The amounts were represented in mg phenolic compound per kg of dry matter

Six fractions of flavonoid compounds were detected in Saffron as follows; naringin, catechin, epicatechin, pyrogallol, luteolin-7-Oglucoside, and apigenin-7O-glucoside, whereas these flavonoids were not detected in the yarrow flower extract. Naringenin (212.36 ± 1.11 µg/g DW), and luteolin (131.16 ± 1.91 µg/g DW), made up the majority of the flavonoid fractions in yarrow flowers. Quercetin (421.51 ± 1.05 µg/g DW), epicatechin (258.11 ± 2.88 µg/g DW), and naringin (117.16 ± 1.22 µg/g DW) were the main flavonoids in the saffron-flower extract.

Hesperidin (8.23 ± 1.18 µg/g DW) and (7.81 ± 1.44 µg/g DW) are once more listed as having a minor flavonoid fraction in the saffron-flowers and yarrows flowers extract, respectively. According to Janicke et al. [29], dietary fiber is a high source of hydroxycinnamic acids, ferulic acid, and p-coumaric acid. These acids may all help explain why fiber has a preventive effect against colon cancer.

P-coumaric acid has been proven to have anti-oxidant, anti-inflammatory, anti-mutagenic, anti-ulcer, and anticancer activities by Pei et al. [30]. Moreover, numerous studies have demonstrated the biological activity of p-coumaric acid as an antioxidant and anti-tumor agent [31, 32].

### Analysis of volatile content using GC-MS

The volatile components of the yarrow and saffron flower extracts were identified in (Table 2) for the first time in this study using headspace GC-MS. Hexanal (16.25%), *β*-Pinene (7.41%), *β*-Myrcene (5.24%), D-Limonene (5.58%), and Caryophyllene (4.38%) were the significant compounds found in yarrows. Conversely, Saffron included considerable amounts of D-limonene (4.89%), isophorone (12.29%), 4-oxy isophorone (8.19%), and safranal (44.84%). Significant levels of safranal, one of the chemicals used to assess the quality of saffron, were discovered in the extracts analyzed [33, 34]. Safranal, with a ratio of 44.84%, was found to be the primary component in saffron flowers. Isophorone received the second majority, with a ratio of 12.29%. There were compounds with antifungal properties, such as D-limonene, 4-oxo isophorone, 4-hydroxy-2,6,6-tri-methyl-3-oxocyclohexa-1,4-diene-carb-aldehyde, and isobutyl phthalate. Additional ingredients include thymoquinone, decanal, neral, 3-phenyl butyric acid, α-Citral, and anethole.

**Table 2:** Volatile content of flower extracts from yarrows and Saffron flower extract determined by the GC-MS apparatus

Yarrows extract				Saffron extract			
Compounds	%	Rt	KI	Compounds	%	Rt	KI
Hexanal	16.25	3.733		*γ*-Crotonolactone	0.97	5.868	
(E)-2-Hexenal	4.87	4.62		Decanal	0.99	7.887	
Heptanal	1.89	5.581		D-Limonene	4.89	8.78	
*α*-Pinene	1.62	6.365		*p*-Ethylvinylbenzene	1.08	10.308	
Benzaldehyde	2.88	7		Isophorone	12.29	10.923	
N-[5-(2-Hydroxyphenyl)-1,3,4-thiadiazol-2-yl] benzamide	0.93	7.195		2,6,6-Trimethylcyclohexa-1,4-dienecarbaldehyde	1.04	11.521	
3-Carene	0.79	7.321		4-Oxoisophorone	8.19	12.162	
*β*-Pinene	7.41	7.424		Lanierone	2.18	12.66	
6-Methyl-5-heptene-2-one	2.92	7.624		7a-Hydroxymintlactone	0.67	12.837	
*β*-Myrcene	5.24	7.744		2,6,6-Trimethyl-1,4-cyclohexanedione	2.52	13.083	
Isothujol	1.26	7.882		Isoxylaldehyde	0.74	13.409	
trans-2-Ethyl-2-hexen-1-ol	1.04	7.939		Safranal	44.84	14.021	
Octanal	1.17	8.042		4-Methyleneisophorone	2.46	14.296	
α-Ocimene	1.1	8.271		2-Hydroxy-4-oxo isophorone	1.81	14.588	
p-Cymene	1.78	8.654		Neral	0.42	14.725	
D-Limonene	5.58	8.763		Octahydro-2 H-chromen-2-one	0.48	14.851	
Eucalyptol	3.8	8.849		*α*-Citral	0.62	15.486	
trans-Sabinene hydrate	1.64	9.575		Anethole	0.23	15.904	
7-Methyl-3-octyne	1.09	9.884		tert-Butyl-p-benzoquinone	3.3	16.568	
p-Ethylstyrene	0.84	10.302		(6-Hydroxymethyl-2,3-dimethyl phenyl)methanol	0.28	16.768 16.997	
5-Ethyl-5-methyl-2-phenyl-2-oxazoline	0.68	10.519		Triophene-2-thiol, 2-methyl propyl ether	1.26	17.106	
Linalool	0.72	10.691					
Nonanal	3.19	10.805		3,5,5-Trimethyl-4-oxo-2-cyclohexene-1-yl acetate	0.28	17.352 17.666	
2,6-Dimethylcyclohexanol	1.49	10.971		1,2-Diacetin	0.58	18.05	
3-Methylindene	0.87	11.103					
Isophorone	0.79	11.315		Thymoquinone	0.39	18.748	
4-Oxoisophorone	0.65	11.944		4-Hydroxy-2,6,6-trimethyl-3-oxocyclohexa-1,4-dienecarbaldehyde	3.99	18.811 19.469	
Borneol	1.54	12.219		1-Heptadec-1-ynyl-cyclopentanol	0.31	19.623	
Terpinene-4-ol	0.74	12.602					
α-Terpineol	1.56	12.911		4-Hydroxy-β-cyclocitral	0.97	29.494	
Estragole	1.26	13.277		3-Phenylbutyric acid	0.36	38.638	
Safranal	1.84	13.472		7a-Methyl-3-methylene-hexahydro benzofuran-2-one	0.34	40.349 5.868	
Decanal	1.37	13.535		Isobutyl phthalate	0.42	7.887	
Cuminaldehyde	2.06	13.638					
3-Methyl-3-(4-methyl-3-pentenyl)-2-oxiranecarbaldehyd	2.8	14.147		Bis(2-Ethylhexyl) phthalate	0.17	8.78	
(-)-Carvone	1.06	14.645		1,4-Benzenedicarboxylic acid, bis(2-Ethylhexyl)ester	0.93	10.308	
Citral	0.64	14.748					
Anethole	0.8	15.44					
Copaene	0.89	15.87					
Caryophyllene	4.38	18.307					
trans-β-Ionone	0.4	19.125					
*α*-Bulnesene	0.8	21.054					
*α*-Muurolene	0.43	21.323					
*γ*-Cadinene	0.83	21.42					
*δ*-Cadinene	1.2	21.775					
Dihydroactinidiolide	0.57	21.981					
Caryophyllene oxide	0.49	22.175					
Viridiflorol	1.11	23.457					
Isoaromadendrene epoxide	0.77	23.657					

KI: the Kovats Retention Indices were calculated from our analysis concerning a series of n-alkenes; %: Percentage composition of a compound in the analysis materials

The two classes of volatile saffron constituents are separated based on their structural characteristics and/or antecedents. With structures differing from safranal, the first category of compounds includes isophorone and 4-Oxoisophorone. The second category, known as C13-nor isoprenoids, continues from the destruction of lipophilic carotenoids and involves constituents with a partly unsaturated 4-hydroxy-2,6,6-trimethyl-3-oxocyclohexa-1,4-dienecarbaldehyde and an isomer of safranal (2,6,6-trimethyl-1, 4-cyclohexadiene-1-carboxaldehyde) [35–38]. Isophorone, which is also included in saffron, is essential for the aging process because it helps create new compounds [34].

The amount of the safranal compound determines the Saffron’s quality and gives its distinctive aroma. As found by GC-MS in earlier studies, safranal is a crucial key odorant in Saffron. Saffron quality is categorized by its safranal content under ISO standard 3632 [33]. However, as the samples were not standardized concerning the technique of harvesting and the duration of storage, the safranal determination cannot be used to draw any valid conclusions [39].

### Characteristics of the nanoemulsions

The stability and initial properties of an emulsion must be maintained once it has been made for it to be used. Various destabilization mechanisms, such as creaming, flocculation, and coalescence, can cause emulsions to disintegrate [31]. Numerous approaches to examining the stability of emulsions are suggested in the international literature [35–38]. Unfortunately, this instability impacts how they are stored because they tend to separate and degrade. Estimating the dispersed-phase droplet size density, pH measurement, and optical observation are a few of these.

Particle size, zeta potential, viscosity, pH levels, and acidity were used to describe the prepared emulsions of the plant-flower extracts. Table 3 lists the qualities of emulsions made for yarrow and Saffron extracts taken from their flowers. Due to zeta potential values of (-16.31 ± 2.54) and (-18.55 ± 2.31) mV for the yarrow and saffron extracts, respectively, the measured values exhibit greater stability. The saffron emulsion’s properties are better, as evidenced by the polydispersity index (PDI) value being lower (0.34 ± 0.05) than that of the yarrow emulsion (0.41 ± 0.09). For the yarrow and saffron extracts, respectively, the estimated acidity was recorded at 0.25 g citric acid/L of emulsion and 0.34 g citric acid/L of emulsion, both of which were slightly acidic based on the measured values of both emulsions, both of which had pH values that were less than 7. The nanoemulsion of yarrow and saffron extracts exhibits high stability and resists agglomeration, according to the results of the zeta potential analysis. The zeta potential values of the extracts, as shown in Table 3, are within the normal distribution curve, indicating that the synthesized nanoemulsions are largely monodisperse.

**Table 3:** Characteristics of prepared nanoemulsions of yarrow and Saffron extracts from the flowers

Sample	Droplet size (nm)	Zeta potential (mV)	PDI	Viscosity mean (mPa/sec)	pH	Acidity (g citric/L)
**Yarrow**	151.21 ± 1.12	− 16.31 ± 2.54	0.41 ± 0.09	1.41 ± 0.022	6.88 ± 0.11	0.25 ± 0.02
**Saffron**	121.64 ± 2.18	− 18.55 ± 2.31	0.34 ± 0.05	1.26 ± 0.045	6.21 ± 0.27	0.34 ± 0.07

PDI: poly dispersing index; Results were expressed in mean ± SD (n = 3, P < 0.05)

### Cytotoxic activity of yarrow and Saffron

A cell-line assay was used to evaluate the nanoemulsions’ safety characteristics. The in vitro cytotoxic activity of plant flower extracts and their nanoemulsions on HeLa and THLE2 cell lines was examined in comparison to 5-fluorouracil (5-FU) as a reference drug using the MTT (tetrazolium bromide solution) viability assay. This study aimed to test whether 5-fluorouracil was more effective at lowering cell viability than plant flower extracts and their nanoemulsions.

The information in Table 4 displays the outcome of cytotoxicity testing using the MTT assay; values of the IC_50_ against tested solutions made from yarrow and saffron flowers were applied to two different types of health cell lines, and they were noted as having safety properties. The saffron solution’s high safety level was determined to be 1921.37 ± 8.15 µg/mL for the nanoemulsion and 1809.18 ± 7.66 µg/mL for the flower extract. Compared to the reference drug 5-fluorouracil, all tested materials demonstrated safe characteristics.

**Table 4:** Cytotoxic activity of yarrow and saffron flower extracts against HeLa and NHDF cell line using MTT assay

Extract	Cell lines	IC_50_ (µg/ml)
**5-fluorouracil**	HeLa	70.69 ± 2.69
	NHDF	72.08 ± 1.47
**Yarrow nanoemulsion (MTT)**	HeLa	527.42 ± 5.71
	NHDF	543.29 ± 4.88
**Yarrow extract (MTT)**	HeLa	614.33 ± 6.22
	NHDF	637.11 ± 5.91
**Saffron nanoemulsion (MTT)**	HeLa	1857.24 ± 7.28
	NHDF	1921.37 ± 8.15
**Saffron extract (MTT)**	HeLa	1809.18 ± 7.66
	NHDF	1846.54 ± 6.94

Results were expressed in mean ± SD (n = 3, P < 0.05). MTT: tetrazolium bromide solution

Numerous in vitro and in vivo models have been used to investigate the cytotoxic activity of yarrow and saffron extracts. According to the findings of these investigations, yarrow and Saffron both exhibit cytotoxic activity against a number of cancer cell lines, including lung, breast, ovarian, prostate, and colon cancer cells [40]. Flavonoids, terpenoids, and sesquiterpene lactones, among other chemicals, have been implicated in the cytotoxic activity of yarrow. These substances have been demonstrated to stop the development of cancer cells by causing apoptosis (cell death) and preventing angiogenesis (the growth of new blood vessels) in the tumor [41]. It is significant to note that the cytotoxic activity of these extracts has been investigated in vitro and in vivo models, and further study is required to confirm the findings and determine the most effective way to use these extracts in the treatment of cancer.

### Antibacterial effect of the extracts and their nanoemulsions

Saffron and yarrow extracts and their nanoemulsions were tested for their antibacterial effects on Gram-positive and Gram-negative pathogenic strains. The inhibition zone produced by applying saffron extract was more effective than that produced by evaluating yarrow extract (Table 5). Against the strain of *Staphylococcus aureus* ATCC 13565, the saffron extract showed the highest level of inhibition at 14.21 ± 1.07 mm of inhibition zone diameter. Saffron application in a nanoemulsion increased this value to 18.34 ± 1.21 mm of inhibition zone diameter. Once more, yarrow’s inhibition was improved by its assessment in nanoemulsion form. Furthermore, the inhibition zones recorded against the gram-negative pathogenic bacterial strains were observed to be smaller than those recorded against the Gram-positive bacterial strains. The higher cell wall resistance in Gram-negative bacterial strains is responsible for this inhibition lowering.

**Table 5:** Antibacterial effect of yarrow and saffron extracts and their nanoemulsions against pathogenic bacteria strains

	Yarrow	Saffron	Nano-yarrow	Nano-saffron
**Gram-positive**	**IZD (mm)**			
*Staphylococcus aureus* ATCC 13565	11.19 ± 1.05	14.21 ± 1.07	12.66 ± 1.31	18.34 ± 1.21
*Bacillus cereus* EMCC1080	10.27 ± 1.31	13.08 ± 1.22	11.74 ± 2.08	19.63 ± 1.05
*Listeria monocytogenes* ATCC 19111	10.81 ± 1.56	13.48 ± 1.87	11.64 ± 1.15	19.37 ± 1.08
*Enterococcus faecalis* ATCC 33186	11.02 ± 2.05	14.02 ± 2.18	11.87 ± 1.81	19.51 ± 1.37
**Gram-negative**	**IZD (mm)**			
*Escherichia coli* ATCC 51659	7.27 ± 1.05	12.08 ± 1.02	10.05 ± 1.18	17.71 ± 1.27
*Klebsiella pneumonia* LMD 7726	5.19 ± 1.27	10.56 ± 1.05	9.94 ± 1.56	17.91 ± 1.07
*Pseudomonas aeruginosa* NRRL B-272	5.02 ± 1.02	11.54 ± 1.27	10.82 ± 1.88	16.88 ± 1.02
*Salmonella enterica* ATCC 10708	4.91 ± 1.11	11.27 ± 1.34	10.64 ± 2.02	16.94 ± 1.18

The data were expressed as means ± SD (where n = 3). IZD: inhibition zone diameter measured in millimeters

Researchers have looked into the antagonistic activity of the upper parts of the *Achillea clavennae*, *Achillea holosericea*, *Achillea lingulate*, and *Achillea millefolium* extracts against five bacteria (*Staphylococcus aureus*, *Escherichia coli*, *Klebsiella pneumoniae*, *Pseudomonas aeruginosa*, and *Salmonella enteritidis*) and two fungi. All four species’ extracts demonstrated a wide range of antimicrobial activity against every tested strain [42]. Still, a variety of theories have been advanced to explain the antibacterial action of nanoemulsions, such as (1) the generation of reactive oxygen species and (2) the attachment of nanoparticles to bacteria and subsequent injury to the bacteria [43–45].

Previous studies pointed out the impact of natural extract as an antimicrobial agent against harmful microorganisms [46]. Numerous published research on the antibacterial activity of nanoemulsions against Gram-positive and Gram-negative bacteria [47, 48], revealed that nanoemulsions had very little antibacterial impact on gram-positive bacteria. It’s interesting to note that both gram-positive and gram-negative bacteria were successfully suppressed by yarrow and saffron flower nanoemulsions, indicating that the variation in the bacterial wall does not impact the antibacterial activity of nanoemulsions.

### Antifungal effect of the extracts and their nanoemulsions

Fewer inhibition zones were recorded against fungal strains than against pathogenic bacterial strains. Applying saffron nanoemulsion resulted in a higher inhibition zone being recorded against the *Fusarium* fungus strain (Table 6). The saffron extract nanoemulsion was the most potent antifungal treatment among the tested extracts of yarrow, Saffron, and their nanoemulsions. It has been observed that Saffron (extract or nanoemulsion) exhibits antifungal effectiveness that is even higher than yarrow nanoemulsion. The effect of saffron nanoemulsion on the examined mycotoxigenic fungi strains was also considered. According to our research, the nanodroplet size and combination of bioactive ingredients in the yarrow and saffron extracts affect the antifungal activity. These outcomes are in line with earlier reports [49–51].

**Table 6:** Antifungal impact of yarrow and Saffron extracts and their nanoemulsions against harmful fungal strains

Strain	Yarrow	Saffron	Nano- Yarrow	Nano-Saffron
*Candida albicans* ATCC 18804	4.67 ± 1.06	7.05 ± 1.02	5.02 ± 1.18	7.91 ± 1.02
* A. flavus* ITEM 698	3.89 ± 0.56	5.81 ± 0.59	4.28 ± 1.05	6.54 ± 1.77
* A. parasiticus* ITEM 11	4.37 ± 1.51	6.94 ± 0.88	5.31 ± 1.21	8.12 ± 0.88
*P. verrucosum* NRRL 695	5.02 ± 1.21	7.37 ± 0.91	6.27 ± 1.55	7.98 ± 1.36
* F. graminearum* ATCC 56091	5.88 ± 1.65	8.97 ± 0.74	7.05 ± 1.81	10.26 ± 1.24

The data were expressed as means ± SD (where n = 3)

### Simulated anti-aflatoxigenic impact

Simulated media was used to assess the influence of applied extracts and nanoemulsions against two species of aflatoxigenic fungi (*A. flavus* ITEM 698 and *A. parasiticus* ITEM 11) Table 7. The outcomes reflect an inhibition influence of nano-Saffron, reaching 69.64% and 71.9% for *A. flavus* and *A. parasiticus*, respectively. These ratios of inhibition for the fungal growth were 40.03% and 34.06% using nano-yarrow extract for *A. flavus* and *A. parasiticus* growth, respectively. The nanoemulsion of the two flower extracts from yarrow and Saffron was recorded as more effective in the fungal growth reduction than the standard extracts.

**Table 7:** The efficacy of extracts and nanoemulsions prepared from yarrow and Saffron extracts against the growth of *A. flavus* and *A. parasiticus* toxigenic strains in simulated liquid media

*Concentrations*	*A. flavus* *(g)*	%	*A. parasiticus* *(g)*	*%*
**Control**	6.2188	0	5.6761	0
**Yarrow**	4.0151	35.44	4.0050	29.44
**Saffron**	2.2328	64.09	1.9910	64.92
**Nano-yarrow**	3.7291	40.03	3.7425	34.06
**Nano-saffron**	1.8881	69.64	1.5951	71.90

The influences of the extracted yarrow and Saffron and its produced nanoemulsion by carboxymethyl-cellulose and Arabic gum on the mycelia growth of *A. flavus* and *A. parasiticus* during five days of incubation (22 ± 2 ^o^C) displayed that the control plate qualified fast proliferation. In dissimilarity, the plates containing yarrow and Saffron and their organized nanoemulsion considerably (p < 0.05) inhibited mycelial proliferation [52], which agreed with the outcomes of the existing study. According to the obtained data, the Saffron extract and its nanoemulsion confirmed greater fungicidal efficacy than the yarrow extract and its nanoemulsion. It seems that the connected nanoemulsion restricts the budding process of fungi, killing off the isolates in the process. The outcomes displayed that as they affected the mycelial growth, they also affected aflatoxin secretion. The early outcomes are stated by Tondervik et al. [53], who displayed the aptitude of sodium alginate to stop the fungal cell growth of *Candida* and *Aspergillus* spp. [54].

### Effect of extracts and nanoemulsion on the reduction of aflatoxins production

When compared to the control, a reduction in aflatoxin indicates the effectiveness of yarrow and saffron flower extracts and their nanoemulsions against the producing strain of *A. flavus.* AFB_1_, AFB_2_, AFG_1_, and AFG_2_ aflatoxins concentrations were measured in the liquid media containing yeast extract sucrose in both the treated media, which also contained fungal spores and tested extract, and the controlled media, which only contained fungal spores. The data in Table 8 exhibited that the saffron flower extract nanoemulsion was the most effective treatment for reducing AFs, followed by the saffron flower extract. The reduction ratio of AFs using saffron-flower extract nanoemulsion ranged from 68.5% for AFB_1_ to 59.8% for AFG_2_. The reduction was accomplished with yarrow extract, and although its nanoemulsion had lower effectiveness than saffron extracts, it was still efficacious.

**Table 8:** Anti-aflatoxigenic impact of yarrow and saffron flower extracts and their nanoemulsions to reduce aflatoxin production in liquid media

*Concentrations*	AFB_1_	AFB_2_	AFG_1_	AFG_2_
**Control**	265.21 ± 2.47	190.33 ± 1.81	212.63 ± 2.58	177.28 ± 2.51
**Yarrow**	134.21 ± 2.34	127.13 ± 1.97	114.21 ± 2.08	110.66 ± 2.19
**Saffron**	98.55 ± 2.67	81.41 ± 2.05	90.61 ± 3.51	80.82 ± 3.57
**Nano-**y**arrow**	118.16 ± 1.56	114.56 ± 2.47	106.18 ± 1.66	102.66 ± 1.49
**Nano-saffron**	83.41 ± 2.05	75.37 ± 1.02	77.42 ± 1.58	71.22 ± 1.64

The data were expressed as means ± SD (where n = 3)

The antifungal effect and inhibition of aflatoxin secretion for yarrow and saffron are well-mentioned in the literature because of its bioactive ingredients, particularly Hexanal (16.25%), *β*-Pinene (7.41%), *β*-Myrcene (5.24%), D-Limonene (5.58%) and caryophyllene (4.38%) for yarrow and D-Limonene (4.89%); Isophorone (12.29%); 4-Oxoisophorone (8.19%); Safranal (44.84%) for Saffron. These outcomes agree with the current study’s findings [33, 34, 42, 45, 55].

The effectiveness of this composite at releasing yarrow, Saffron, and their nanoemulsion material during the period when spores are incubating in the growth media may help to explain this effect. In addition, particle size and zeta-potential values may also improve emulsion diffusion through the fungal cell wall and impact metabolic processes [56]. The findings might imply that the action of yarrow and Saffron ingredients in reducing the secretion of aflatoxins is related to the interactions with the primary enzymes implicated in mycotoxin secretion.

### Application of nanoemulsions in fruit preservation

**Fig. 2:**
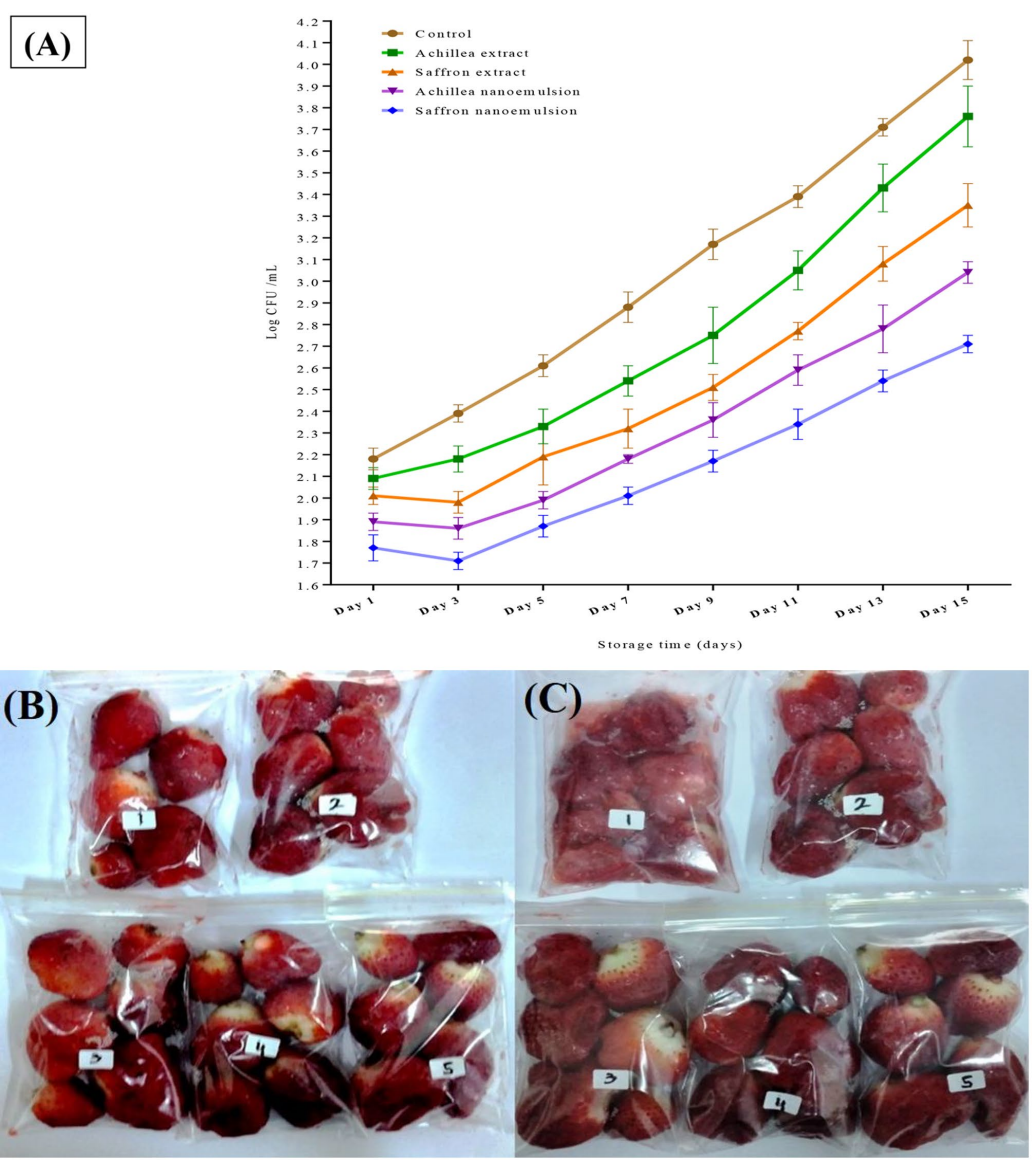
**(A)** The changes recorded in the *Aspergillus* log CFU during the cold storage of infected strawberry fruits, **(B)** Illustrated pictures regarding the effect of extracts or nanoemulsions of yarrow (*Achillea millefolium*) and saffron (*Crocus sativus*) at zero time, and **(C)** Illustrated pictures regarding the effect of extracts or nanoemulsions of yarrow and saffron at storage time (15 days) (1) Control untreated strawberry fruits, (2) treated with yarrow extract, (3) treated with saffron extract, (4) treated with yarrow nanoemulsion, and (5) treated with saffron nanoemulsion

Saffron nanoemulsion was the most efficient substance, according to experiments using yarrow and saffron and their nanoemulsions to coat the strawberry fruits during their cold storage as shown in Fig. 2. After inoculating *Aspergillus* spores, the log CFU increment rate varied between the various coating solutions applied to the fruits. The viability of the fungal spores is recorded for nanoemulsion application as less than for their corresponding extracts (Fig. 2A). These findings demonstrate how nanoemulsions can preserve the trace elements of an applied extract and have a greater antioxidant effect, which helps slow the fungal growth rate. However, The control fruits showed signs of decay in the capture after storage; whereas, the coated fruits, whether by extracts or nanoemulsion, are still distinct (Fig. 2B&C). Fruit groups coated with yarrow extract show few signs of decay. The yarrow nanoemulsion-coated fruits do not exhibit this decay (Fig. 2B&C).

Another thing to note is that strawberries coated in saffron nanoemulsion are thought to have the lowest fungal load of any fruit, which may indicate greater resistance to the growth of fungi that would otherwise shorten their shelf life (Fig. 2B&C). This strategy is comparable to researching how an antimicrobial edible chitosan coating affects the quality, safety, and sensory attributes of chilled strawberries, which aligns with the current study’s findings [57, 58]. The results showed that coating fresh-cut strawberries with nanoemulsions of yarrow and saffron improved their quality and shelf life. These results are in accordance with the previous investigations that referred to the essential compounds and bioactive constituents in food safety valorization [59, 60].

## Conclusion

Yarrow and saffron flower extracts and their nanoemulsions have been shown in this study to have antimycotic and anti-aflatoxigenic properties. According to these findings, these extracts and nanoemulsions may be used as natural preservatives in food products to stop fungal development and aflatoxin contamination. By preventing the growth of fungi and minimizing the production of aflatoxins, strawberries coated with yarrow and saffron flower extracts and their nanoemulsions may be able to last longer on store shelves. The nanoemulsion form might also improve the antimicrobial capabilities of the extracts. These results imply that coating strawberries with these nanoemulsions and extracts may successfully preserve the fruit and guarantee food safety. Additional study is most likely required to verify these results and determine the most effective ways to use the extracts and nanoemulsions on strawberries.

## Data Availability

The datasets used and/or analyzed during the current study are available from the corresponding author upon reasonable request.
